# Teaching Under Fire: A Cross-Sectional Study of Dispositional and Life-Stage Correlates of Perceived Resilience Among Israeli Educators Under Missile Threat

**DOI:** 10.3390/bs16091615

**Published:** 2026-09-10

**Authors:** Chen Hanna Ryder, Carmit Gal, Revital Hamerman, Ester Halfon, Samih Badarny, Rema Nassar, Yazid Badarny

**Affiliations:** 1 Brain and Behavior Research Institute, Western Galilee Academic College, Acre 2412101, Israel; chenr@wgalil.ac.il (C.H.R.); carmitg@wgalil.ac.il (C.G.); revital@kramim.ort.org.il (R.H.); 2 Teacher Education Department, Shaanan College of Education, Haifa 2623607, Israel; halfonester@shaanan.ac.il; 3 Neurology Department, Galilee Medical Center, Nahariya 2210001, Israel; 4 Azrieli Faculty of Medicine, Bar-Ilan University, Safed 1311502, Israel; 5 Department of Surgery, Bnai Zion Medical Center, Haifa 3339473, Israel; rima.nassar@b-zion.org.il; 6 Department of Neurosurgery, Rambam Medical Center, Haifa 3109601, Israel; y_badarny@rambam.health.gov.il

**Keywords:** perceived resilience, psychological resilience, Big Five personality, emotional stability, conscientiousness, life-stage correlates, conservation of resources theory, armed conflict, cross-sectional study, Israel

## Abstract

Educators who keep teaching under wartime missile threat occupy a demanding dual role, sustaining instruction and students’ emotional needs while personally exposed. This descriptive cross-sectional study examined dispositional and life-stage correlates of perceived resilience among 234 educators in Northern Israel during active missile threat (April–July 2024); all shared the same regional context, though individual exposure was unmeasured. Participants completed the CD-RISC-10 and the Ten-Item Personality Inventory. Two confirmatory questions concerned personality–resilience associations; a third, exploratory question concerned the parent–non-parent contrast. In a hierarchical regression, Emotional Stability (β = 0.37) and Conscientiousness (β = 0.24) were the strongest observed dispositional correlates, and the model accounted for 41.0% of the variance; because Emotional Stability and the resilience measure overlap conceptually and in method, it is reported as an observed correlate rather than a predictor. Parents scored higher than non-parents (d = 0.68), but minimal covariate overlap (only 27 of 186 parents matchable) means parenthood cannot be separated from an incompletely measured life-stage configuration; it is retained only as an exploratory marker. Interpreted within a Conservation of Resources framework, this study offers a contextual extension of established personality–resilience findings to educators under sustained missile threat; all findings are associational.

## 1. Introduction

Educators’ capacity to keep teaching effectively under adversity depends in large part on their resilience, their perceived capacity to function under pressure, which reflects the interplay of dispositional traits, accumulated life experience, relational ties, and the intensity of the stressors they face (Bonanno, 2004; Folkman, 2008; Hobfoll, 1989, 2001; Lazarus & Folkman, 1984). Systematic reviews of teacher resilience indicate that self-efficacy, emotion regulation, and professional experience are among the most consistent correlates, alongside dispositional factors, and recent evidence further identifies specific psychological factors contributing to teachers’ resilience (Beltman et al., 2011; Lu et al., 2024; Munezero et al., 2026; Wang et al., 2025). A meta-analysis of teacher self-efficacy and resilience found a significant positive correlation (r = 0.49) across 21 studies (Mu et al., 2024). Educators working under conflict conditions are reported to show elevated strain and burnout vulnerability, suggesting that emotion regulation and contextual support may serve as key moderators (Jayusi, 2025; Neustadter et al., 2026; Täschner et al., 2025). These findings underscore the importance of examining both dispositional and contextual factors when modeling perceived resilience in educational professionals working in conflict-affected settings.

Crucially, the resources that sustain resilience are not located in the individual alone: they operate across multiple ecological levels, from school climate and educator well-being initiatives to protective factors that extend beyond individual traits (Munezero et al., 2026; Patrick et al., 2024; Ren et al., 2025). Recent systematic review evidence further indicates that teachers’ emotional competence and well-being are supported by psychological resources and by structured interventions targeting emotion regulation, mindfulness, and reflective capacity, although methodological heterogeneity still limits strong generalization (Cervellione et al., 2025).

This resilience is consistently linked to greater professional well-being and to lower burnout; yet, as recent teacher-focused studies emphasize, it should not be collapsed conceptually into those broader outcomes (Çiçek et al., 2026; Pöysä et al., 2026; Yang et al., 2025). This distinction is especially important in the present manuscript, which examines perceived resilience specifically rather than overall teacher well-being, occupational functioning, or intervention response.

In the present study, perceived resilience is operationalized as self-reported coping capacity, as assessed by the CD-RISC-10, rather than as observed behavioral adaptation, trauma symptom reduction, or occupational performance (Bonanno, 2004; Campbell-Sills & Stein, 2007; Connor & Davidson, 2003). It is important to distinguish this outcome from related but distinct constructs: teacher resilience (a process-oriented construct), occupational well-being (a broader state), burnout (resource depletion), and stress appraisal (a cognitive evaluation process). The CD-RISC-10 captures how capable an individual perceives themselves to be under pressure, an outcome that can be empirically observed without requiring causal inference about its antecedents. Psychometric reviews confirm the CD-RISC-10’s strong cross-cultural validity (Flores-Buils et al., 2022; Sharif-Nia et al., 2024).

Between April and July 2024, educators in Northern Israel continued teaching under active wartime missile threat while navigating disruption, uncertainty, and repeated safety demands affecting their schools, households, and local communities. In such conditions, educators occupy a distinctive psychological position: they remain responsible for instructional continuity and for students’ emotional containment, a role that gains particular salience given the well-documented psychological effects of war on children (Slone & Mann, 2016) and the broader challenges educators face in addressing situations that threaten pupils’ well-being (Salo & Kajamies, 2024), while simultaneously functioning within the same danger environment as the families they serve. This dual status makes them an informative occupational group for studying perceived resilience under chronic threat, especially in settings where professional role demands and the surrounding threat context are tightly intertwined (Baum, 2012; Nuttman-Shwartz, 2015; Tosone et al., 2012).

Recent empirical work from conflict-affected educational settings underscores the importance of examining educators as a distinct wartime population rather than treating them only as a generic helping profession. Studies conducted during the current regional crisis have documented resilience challenges, burnout-related strain, and the relevance of emotion regulation among Israeli educators and related professionals, while research from other war contexts suggests that teachers working under continued threat report high stress, mixed coping patterns, and possible benefits from structured support (Harb et al., 2026; Jayusi, 2025; Nadyukova & Frenzel, 2025; Neustadter et al., 2026; Tarrasch & Russo-Netzer, 2026).

Despite their evident relevance, educators who continue working through active conflict remain markedly underrepresented in the resilience literature, which has been built largely on Western, non-conflict samples.

All participants shared a regional wartime context, but individual exposure intensity was not measured; evidence that conflict-related memories vary within affected populations underscores this limitation (Borinca et al., 2026), which is addressed in Section 2.4 and Section 4.5.

Turning from the shared regional context to the individual, meta-analytic evidence identifies Emotional Stability (low Neuroticism) as the strongest dispositional correlate of self-reported resilience across diverse samples and contexts, with Conscientiousness and Extraversion next and comparable in magnitude (Oshio et al., 2018). Luo et al. (2023) report parallel meta-analytic evidence for perceived stress, a related but distinct outcome. Both Emotional Stability and Conscientiousness show mean-level increases across adulthood attributed to social investment in age-normative roles, including parenthood and professional advancement (Bleidorn et al., 2013; Roberts & Mroczek, 2008). On that literature, participants further along this life-stage gradient (parents, who are also older and more experienced) might be expected to score somewhat higher on these traits; this expectation was not tested as a confirmatory hypothesis here, and the observed group differences on the personality indicators were small (Table S1).

Parenthood was examined here as a readily observable life-stage marker rather than as an isolable psychological factor. In this sample, parents were typically older and more experienced than non-parents, and marriage commonly accompanied parenthood, reflecting a coherent life-stage configuration. Examining such an observable marker is descriptively useful for characterizing heterogeneity within the sample; it should not be used to infer psychological need or to target support on the basis of parenthood. Accordingly, parenthood is retained here only as an exploratory demographic marker embedded within a broader life-stage configuration, and this study is framed around descriptive variation across observed life-stage characteristics rather than around a parenthood effect.

The Ten-Item Personality Inventory (TIPI) provides brief, broad indicators of the Big Five domains and is used here to operationalize the focal dispositional correlates (Credé et al., 2012; Gosling et al., 2003).

Against this background, the present study set out to identify which dispositional and life-stage characteristics are associated with perceived resilience among educators who continued teaching under active missile threat. The two sets of characteristics serve complementary purposes rather than competing ones. In the broader literature, personality traits such as Emotional Stability and Conscientiousness are robust dispositional correlates of adaptive functioning under stress; because the present cross-sectional design measured only the traits themselves and not any underlying psychological process, they are treated here as dispositional correlates rather than as measured or modifiable mechanisms. Readily observable life-stage characteristics are retained as descriptive markers of who, within this workforce, reported higher perceived resilience.

These considerations situate the present study within Conservation of Resources (COR) theory, which holds that psychological and social resources jointly shape how individuals withstand stress (Folkman, 2008; Hobfoll, 1989). Under sustained collective threat, the capacity to draw on and protect these resources and to regulate one’s own responses becomes especially consequential for resilience (Hobfoll et al., 2018), and evidence from Israeli samples links Conservation of Resources processes to resilience outcomes under population-level crisis conditions (Egozi Farkash et al., 2022). This framework provides an interpretive context for the observed dispositional and demographic correlates examined here; the underlying psychological and social resources were not measured.

### The Present Study: Research Questions and Analytic Status

This study addressed three research questions. The first two follow directly from the aim stated above and from the meta-analytic literature that motivated the design; the zero-order correlations and the first two steps of the hierarchical model were planned before the data were analyzed and are reported as confirmatory. The third is secondary and exploratory and is labeled as such throughout. This study was not preregistered.

RQ1 (confirmatory): Are Emotional Stability and Conscientiousness positively associated with perceived resilience among educators working under sustained regional missile threat? H1: Both traits will show positive associations with CD-RISC-10 scores.

RQ2 (confirmatory): Do these dispositional associations hold once age and teaching experience are taken into account? H2: The personality block will account for incremental variance in CD-RISC-10 scores beyond the two life-stage covariates entered at Step 1.

RQ3 (exploratory): Do parent and non-parent educators differ in perceived resilience, and can any such difference be distinguished from the correlated life-stage characteristics in which parenthood is embedded? No directional hypothesis was specified. Parenthood was recorded as part of routine sociodemographic background rather than as a planned focus, and the decision to examine it as a life-stage marker followed inspection of the sample’s composition. All analyses bearing on RQ3 (the group contrast, Step 3 of the hierarchical model, the sensitivity ANCOVA, and the matching and overlap diagnostics) are therefore diagnostic rather than confirmatory, and no confirmatory weight is placed on them.

So framed, this study is a contextual extension rather than a new theoretical claim. The personality–resilience associations at issue are well established in the wider literature; what is not established is whether they are also observed among educators who continue working under sustained collective threat, a population that remains underrepresented in that literature. That is the contribution the present design can support.

## 2. Materials and Methods

### 2.1. Design and Ethical Considerations

This was a descriptive cross-sectional observational study designed to characterize observed variation within a naturally occurring wartime field sample rather than to support causal inference. This study’s framing is explicitly comparative–descriptive: it documents group contrasts without claiming to isolate the psychological effect of any single variable. All sample-size decisions, measures, and major analytic choices are reported in accordance with JARS recommendations (Appelbaum et al., 2018) and the STROBE framework for observational studies (von Elm et al., 2008); sensitivity analyses are detailed in the Supplementary Materials. This study was approved by the Institutional Review Board (IRB) of Western Galilee Academic College (Protocol No. IRB-2024-047). Informed consent was obtained electronically from all participants prior to participation. Participation was anonymous and entirely voluntary. Contact information for psychological support services was provided at survey completion to minimize potential distress.

### 2.2. Participants

Participants were 234 educators (classroom teachers, homeroom teachers, and special education staff) who were actively employed and residing in Northern Israel during the data collection period (April–July 2024, the final months of the 2023–2024 academic year). Eligibility required active school employment and Northern Israeli residence during this period. Recruitment was conducted digitally through regional teacher unions and school principals across Northern Israel. Because recruitment was conducted through institutional gatekeepers during an active wartime period, a formal response rate could not be calculated. No automated collection-time safeguards (duplicate-IP screening or completion-time capture) were applied at the point of data collection, as these were impracticable under anonymous field conditions; forced-response item settings were used to minimize missing data. Because forced-response settings required a response to every item before submission, all 234 returned questionnaires were complete, with no missing data on any analyzed variable (0% missingness). To evaluate response quality, we instead conducted post hoc careless-responding screening on the item-level data following established procedures (Curran, 2016; Meade & Craig, 2012), comprising (i) a long string index (the longest run of identical consecutive responses across the 20 CD-RISC-10 and TIPI items), (ii) individual-response variability (the within-person standard deviation across items), and (iii) a psychometric consistency check based on the reverse-keyed TIPI item pairs. Sixteen respondents (6.8%) exceeded at least one predefined threshold (a long string of more than 10 identical consecutive responses, zero within-person variance in the CD-RISC-10, or reverse-keyed inconsistency greater than three scale points on a majority of TIPI domains). All primary analyses were repeated with these cases excluded (n = 218), and the pattern of results was unchanged (Table S7); all 234 cases were therefore retained in the analyses reported below. Of the 234 participants, 186 (79.5%) were parents and 48 (20.5%) were non-parents; 191 (81.6%) were married or cohabiting. The sample was predominantly female (56.8%), with a mean age of 42.5 years (SD = 9.0) and mean teaching experience of 17.4 years (SD = 8.0).

Parenthood was strongly intertwined with broader life-stage differences: parents were substantially older (M = 44.8 vs. 33.7 years; d = 1.41) and more experienced (M = 19.2 vs. 10.6 years; d = 1.18) than non-parents, and marital status overlapped meaningfully with parenthood (Cramér’s V = 0.33). School-level clustering could not be modeled because school identifiers were not collected (Raudenbush & Bryk, 2002). A full summary of participant characteristics by parenthood status is presented in Table 1.

### 2.3. Sample Size Determination

The sample-size rationale was anchored to the smallest theoretically meaningful focal association rather than to an assumed medium omnibus regression effect. The focal associations are the personality–resilience correlations, for which robust meta-analytic benchmarks are available: in the work of Oshio et al. (2018; k = 30, N = 15,609), resilience correlated r = 0.46 with Emotional Stability (reverse-scored Neuroticism) and r = 0.42 with Conscientiousness, and the smallest Big Five–resilience correlation was r = 0.31 (Agreeableness). We adopted the smallest of these (r ≈ 0.30) as the smallest effect this study should be able to detect. For a bivariate correlation with the obtained sample (N = 234, α = 0.05, two-tailed, power = 0.80), the minimum detectable correlation is r = 0.18 (Cohen, 1988), computed with G*Power version 3.1.9.7 (Faul et al., 2007; Heinrich Heine University Düsseldorf, Düsseldorf, Germany). This minimum lies well below the r ≈ 0.30 benchmark and far below the focal Emotional Stability (r ≈ 0.46) and Conscientiousness (r ≈ 0.42) correlations, indicating that the obtained sample is adequately powered to detect even the weakest theoretically meaningful trait–resilience association. For the hierarchical model, a sensitivity analysis for the incremental contribution of one predictor entered at the final step indicated a minimum detectable effect of f^2^ = 0.034 at 80% power and α = 0.05. The observed increment associated with the exploratory parenthood term was ΔR^2^ = 0.035, corresponding to f^2^ = 0.059, and therefore exceeded this threshold. The Step 2 increment, ΔR^2^ = 0.236, is not compared with this threshold, which was computed for a single predictor rather than for a five-predictor block. Because the parent–non-parent comparison is exploratory and the groups are of markedly unequal size (n_1_ = 186, n_2_ = 48), a sensitivity analysis indicated a minimum detectable effect of d = 0.46 for Welch’s *t*-test (Faul et al., 2007) (power = 0.80, α = 0.05); the observed unadjusted difference (d = 0.68) exceeded this threshold but is interpreted only descriptively given the limited covariate overlap between groups (see Section 2.6 and Table S1).

### 2.4. Procedure

Given ethical concerns about participant burden during active conflict, no direct individual-level measure of war-exposure severity was collected; this decision was approved under Protocol No. IRB-2024-047. All participants lived and worked in Northern Israel during the same period of intensive missile threat, but exposure intensity was not assumed to be uniform. The consequences of this unmeasured within-context variation are addressed in Section 4.5.

The CD-RISC-10 was administered using Hebrew-language materials available to the research team through the library services of Western Galilee Academic College. Written permission for use of the Hebrew CD-RISC-10 was obtained from the copyright holder of the scale and is available from the corresponding author upon request; no CD-RISC items are reproduced in this manuscript or in the Supplementary Materials. The TIPI was likewise administered in Hebrew so that all study instruments were completed in Hebrew, the survey administration language used in the present study. Because measurement invariance was not formally tested (Section 4.5), observed-score differences are interpreted as manifest-score associations rather than as confirmed latent-construct differences.

### 2.5. Measures

*Perceived Psychological Resilience (CD-RISC-10):* The 10-item Connor–Davidson Resilience Scale (CD-RISC-10) (Campbell-Sills & Stein, 2007; Connor & Davidson, 2003) assessed self-reported general psychological resilience on a 5-point Likert scale (0 = not true at all; 4 = true nearly all the time). Scores were computed as mean item scores (range 0–4); higher scores indicate greater perceived resilience. The instrument was treated as a measure of perceived resilience, not as an index of trauma symptom burden, stress appraisal, or coping strategy use. Internal consistency was high (α = 0.97); because this value is unusually high for a 10-item scale, it was interpreted cautiously as potentially reflecting strong item homogeneity alongside possible redundancy or shared method variance (Podsakoff et al., 2003). To characterize that homogeneity directly, the average inter-item correlation was computed directly from the ten-item correlation matrix and was r = 0.77, consistent with the value the standardized-alpha identity r = α/[k − α(k − 1)] returns for k = 10 at this level of α. A mean inter-item correlation of this magnitude lies far above the 0.15–0.50 band conventionally taken to indicate adequate but non-redundant item coverage (Clark & Watson, 1995), indicating that the ten items functioned as near-parallel indicators of a single narrow content domain. McDonald’s ω was 0.97. Together with α = 0.97 and the average inter-item correlation of r = 0.77 reported above, this indicates high internal consistency, together with substantial item overlap; the average inter-item correlation is the index that speaks to the overlap directly. A one-factor confirmatory factor analysis of the ten items fitted the data well, χ^2^(35) = 46.01, *p* = 0.101, CFI = 0.996, TLI = 0.995, RMSEA = 0.037, SRMR = 0.013, with standardized loadings between 0.84 and 0.91, supporting the unidimensional mean-score scoring used here (Table S8). A psychometric review using COSMIN methodology confirmed the CD-RISC’s strong cross-cultural validity across multiple language and population groups (Sharif-Nia et al., 2024). A validation study of the Spanish version among teachers confirmed adequate psychometric properties for educator populations specifically (Flores-Buils et al., 2022).

*Big Five Personality Indicators (TIPI):* The Ten-Item Personality Inventory (TIPI) (Gosling et al., 2003) served as a brief measure of five broad dispositional domains, providing two-item indicators of Extraversion, Agreeableness, Conscientiousness, Emotional Stability, and Openness to Experience on a 7-point Likert scale (1 = disagree strongly; 7 = agree strongly). Negatively keyed items were reverse-coded prior to averaging the two items within each Big Five domain. The TIPI was included to operationalize the focal dispositional correlates, not as a precise Big Five assessment. Two-item scales yield reliabilities well below conventional thresholds, and, in this sample, three of the five domains fell below 0.70 (Table 2). Two consequences are carried through the interpretation: coefficients for the low-reliability domains are attenuated toward zero, and so their magnitudes understate any true association; and non-significant coefficients for those domains are treated as uninformative rather than as evidence of no association, since measurement error is at least as plausible an explanation as a true null (Credé et al., 2012). Spearman–Brown split-half reliability estimates for the present sample are reported in Table 2.

### 2.6. Analytic Strategy

The primary analytic model was a hierarchical multiple regression in which age and teaching experience were entered first (Step 1), followed by the Big Five personality indicators as focal dispositional indicators (Step 2), and finally parenthood status as an associated demographic characteristic (Step 3). This entry order was theory-guided: age and experience were entered first to account for life-stage variation, personality indicators were then added to estimate incremental dispositional variance, and parenthood status was entered last to examine whether a statistically detectable residual association remained.

All models reported here were re-estimated for this revision from a single analysis file so that the zero-order correlations, the hierarchical models and the sensitivity analyses all derive from one run. The Supplementary Materials accompanying this revision were generated from that same analysis file and from the present version of this manuscript so that all reported values, sample sizes, table numbering and the manuscript title correspond across the two documents.

Participant sex was recorded but was not included in the focal primary specification, which was built to represent one conceptual structure: dispositional traits together with the age-graded demographic configuration in which parenthood sits. Sex showed no detectable association with parenthood status in this sample (χ^2^(1) = 0.18, *p* = 0.675; Table 1). Because sex may nevertheless be associated with self-reported resilience and with occupational experience, the final model was re-estimated with sex included as a sensitivity analysis (Section 3.5; Table S10).

Step 3 is presented as an exploratory adjusted analysis, not as a demonstration that parenthood is separable from the broader life-stage configuration. Given the severe covariate imbalance between groups (pre-matching standardized mean differences of 1.42 for age and 1.25 for teaching experience), compounded by the 186:48 group-size ratio, only 27 parent educators could be matched 1:1, leaving 159 (85.5%) unmatched (Table S1); covariate adjustment therefore cannot recover exchangeability. The Step 3 coefficient therefore reflects a statistically detectable observed association under severe non-overlap conditions, not a causally identified parenthood effect. Within the present dataset, it should be read as a residual subgroup contrast after partial adjustment rather than evidence of a distinct psychological effect of parenthood. The standardized mean differences reported here and in Table S1 are matching diagnostics computed with the square root of the average of the two group variances in the denominator; they therefore differ slightly from the pooled-standard-deviation effect sizes given for the same variables in Table 1.

Marital status was held out of the primary model for a substantive rather than a statistical reason: it belongs to the same life-stage configuration as parenthood, with which it overlaps in this sample (Cramér’s V = 0.33). Entering both would partition variance within a single conceptual block rather than adjust for a distinct source of confounding, and would make the resulting coefficients harder, not easier, to interpret. Because that is a judgment rather than a necessity, marital status was carried into a sensitivity ANCOVA (Section 3.4; Table S2), in which the parenthood contrast remained directionally similar. Neither specification identifies an independent contribution of either variable; both are read as facets of one incompletely measured configuration. Regression diagnostics confirmed model assumptions were met: residual plots showed no systematic departure from linearity or homoscedasticity, the Shapiro–Wilk test was non-significant (W = 0.990, *p* = 0.094), and Cook’s distance inspection revealed no influential observations (maximum D = 0.08). VIFs did not exceed 3.4 for any predictor, indicating acceptable multicollinearity. Non-parametric robustness testing (Mann–Whitney U; Table S3), propensity-score matching (PSM) as a covariate-overlap diagnostic (Table S1; Ho et al., 2007; Stuart, 2010), and an age-overlap sensitivity analysis (Tables S4 and S5) are reported in full in the Supplementary Materials. Propensity scores were estimated by logistic regression of parenthood status on age, teaching experience, marital status and the five personality indicators, and cases were matched 1:1 by nearest neighbor without replacement within a caliper of 0.20 SD of the logit. All analyses were conducted in IBM SPSS Statistics version 27 (IBM Corp., Armonk, NY, USA), with the exception of the confirmatory factor analysis and McDonald’s ω, which were estimated by maximum likelihood in R version 4.4.1 (R Foundation for Statistical Computing, Vienna, Austria) using the lavaan package (version 0.6-18), and the HC3 heteroscedasticity-consistent standard errors, which were computed in R using the sandwich package (version 3.1-0).

## 3. Results

### 3.1. Descriptive Statistics

Observed CD-RISC-10 scores were moderate overall (M = 2.58, SD = 0.90). Observed scores spanned the full-scale range (minimum = 0.00, maximum = 4.00), with the sample mean falling near the scale midpoint, consistent with meaningful differentiation across the full spectrum of perceived resilience rather than restriction toward the upper bound. As shown in Table 1, parent and non-parent groups differed sharply in age (d = 1.41, *p* < 0.001), teaching experience (d = 1.18, *p* < 0.001), and marital status (Cramér’s V = 0.33, *p* < 0.001) prior to any analytic adjustment. Descriptive statistics for the full sample and personality measures are presented in Table 2. Zero-order correlations among all continuous study variables are presented in Table 3; consistent with meta-analytic estimates for the Big Five and resilience (Oshio et al., 2018), Emotional Stability showed the strongest zero-order association with CD-RISC-10 scores (r = 0.48, *p* < 0.001), followed by Conscientiousness (r = 0.38, *p* < 0.001), while age and teaching experience correlated r = 0.36 and r = 0.35 with resilience, respectively. Age and teaching experience were highly intercorrelated (r = 0.82), consistent with their treatment as interrelated demographic indicators and with the multicollinearity diagnostic summary reported in the Table 4 note. These pre-existing imbalances constitute the central interpretive constraint on all subsequent group comparisons. Any observed difference between groups reflects a compound of correlated life-stage characteristics, not parenthood in isolation. Because age and teaching experience were so strongly correlated (r = 0.82), their individual coefficients in the final model (β = 0.05, *p* = 0.593, and β = 0.13, *p* = 0.155) should not be interpreted as independently established correlates; with overlapping confidence intervals and substantial shared variance, the two predictors are best read as jointly indexing a single dimension of life-stage position rather than as separable effects.

### 3.2. Exploratory Group Contrast

Parents reported higher observed CD-RISC-10 scores (M = 2.70, SD = 0.85) than non-parents (M = 2.11, SD = 0.92): Welch’s t(69.6) = 4.04, *p* < 0.001, d = 0.68, 95% CI [0.36, 1.01]. This constitutes a medium-to-large unadjusted group difference in perceived resilience, representing a raw score difference of 0.59 scale points on a 0–4 metric. The unadjusted contrast is displayed in Figure S1, and a non-parametric Mann–Whitney U test reproduced it (Table S3). Restricting both groups to the overlapping age range of 23–47 years also reproduced it on the reduced sample (n = 160), t(67.4) = 3.25, *p* = 0.002, d = 0.61 (Tables S4 and S5). Because all participants were recruited from the same active-threat regional context, the contrast is not interpretable as exposed versus unexposed. However, the groups were not structurally comparable in age (d = 1.41), teaching experience (d = 1.18), or marital status (V = 0.33), meaning the unadjusted difference reflects the combined influence of correlated life-stage characteristics within a shared wartime context and cannot be attributed to parenthood status alone. This observed variation between subgroups is an exploratory descriptive finding of this study; the mechanisms underlying it remain underdetermined.

### 3.3. Hierarchical Regression

The results of the hierarchical regression predicting CD-RISC-10 scores are presented in Table 4, which reports the complete coefficients, standard errors, confidence intervals, and fit indices for each hierarchical model separately. In Step 1, age and teaching experience together accounted for 14% of variance in perceived resilience, R^2^ = 0.139, F(2, 231) = 18.61, *p* < 0.001. Adding the Big Five personality indicators at Step 2 produced a substantial increment, ΔR^2^ = 0.236, ΔF(5, 226) = 17.03, *p* < 0.001. In the final model, Emotional Stability (β = 0.37, *p* < 0.001) and Conscientiousness (β = 0.24, *p* < 0.001) were the strongest observed dispositional correlates. Because Emotional Stability and the CD-RISC-10 share conceptual content and were measured by the same method within a single session, the Emotional Stability coefficient is reported throughout as the strongest observed correlate rather than as an independent predictor (Section 4).

Adding parenthood status at Step 3 yielded a small but statistically detectable increment, ΔR^2^ = 0.035, ΔF(1, 225) = 13.41, *p* < 0.001. In the final model, the parenthood marker was associated with CD-RISC-10 scores above and beyond age, experience, and all five personality indicators: B = 0.49, SE = 0.13, β = 0.22, t = 3.66, *p* < 0.001, 95% CI [0.23, 0.76]; total model fit: R^2^ = 0.410, adjusted R^2^ = 0.389, F(8, 225) = 19.51, *p* < 0.001. This Step 3 coefficient is reported as part of a secondary, exploratory subgroup analysis conducted under conditions of severe covariate imbalance between parents and non-parents. It does not support causal attribution of the resilience difference to parenthood status, and should be interpreted only as a model-dependent residual association reflecting a compound of unresolved life-stage differences (see Table S1 for covariate-overlap diagnostics). For a single predictor, ΔF equals the square of the corresponding t-statistic; here, the unrounded t = 3.6623 gives t^2^ = 13.41, the reported ΔF. All t, F, ΔF, and confidence interval values were computed from full-precision SPSS output and may therefore differ marginally from values recomputed by hand from the rounded quantities displayed in Table 4. The same applies to the R^2^ and ΔR^2^ values in Table 4: each is computed from the unrounded model fit; so, the displayed increments need not sum exactly to the displayed totals.

Because age and teaching experience were highly collinear (r = 0.82), the parenthood coefficient was re-estimated across three life-stage specifications (age only, teaching experience only, and both covariates together), each additionally including the five personality indicators (Table 5). The parenthood coefficient was stable across specifications (B = 0.49–0.51; β = 0.22–0.23; all *p* < 0.001), indicating that the residual parenthood contrast did not depend on which collinear life-stage covariate was modeled. In contrast, the age and teaching experience coefficients were themselves markedly unstable: each reached significance when entered alone (age β = 0.15, *p* = 0.015; experience β = 0.16, *p* = 0.006) but neither remained significant when both were entered (age β = 0.05, *p* = 0.593; experience β = 0.13, *p* = 0.155). This instability is treated as a substantive finding: age and teaching experience are best interpreted as jointly indexing a single dimension of life-stage position rather than as separable predictors, and no interpretive weight is placed on their individual coefficients.

Because Emotional Stability (the low pole of Neuroticism) and the CD-RISC-10 share conceptual content and were assessed within the same self-report session, their zero-order correlation was examined and the hierarchical model re-estimated with Emotional Stability removed. The two measures correlated r = 0.48 (*p* < 0.001). With Emotional Stability excluded, the model accounted for 29.6% of the variance, a reduction of ΔR^2^ = 0.114 attributable uniquely to Emotional Stability, and the substantive pattern was largely unchanged: Conscientiousness (β = 0.31, *p* < 0.001) and the parenthood marker (B = 0.41, β = 0.19, *p* = 0.005) retained their magnitude and significance. Extraversion, which was non-significant in the full model, reached significance when Emotional Stability was removed (β = 0.13, *p* = 0.031), which is what would be expected of a trait that shares variance with it; age was not significant (β = 0.11, *p* = 0.296) and nor was teaching experience (β = 0.14, *p* = 0.166), consistent with the age–experience collinearity (Table S6). The substantive conclusions therefore do not depend on Emotional Stability, although its unique contribution should be read with construct and method overlap in mind (see Section 4).

### 3.4. Sensitivity ANCOVA

**Figure 1:**
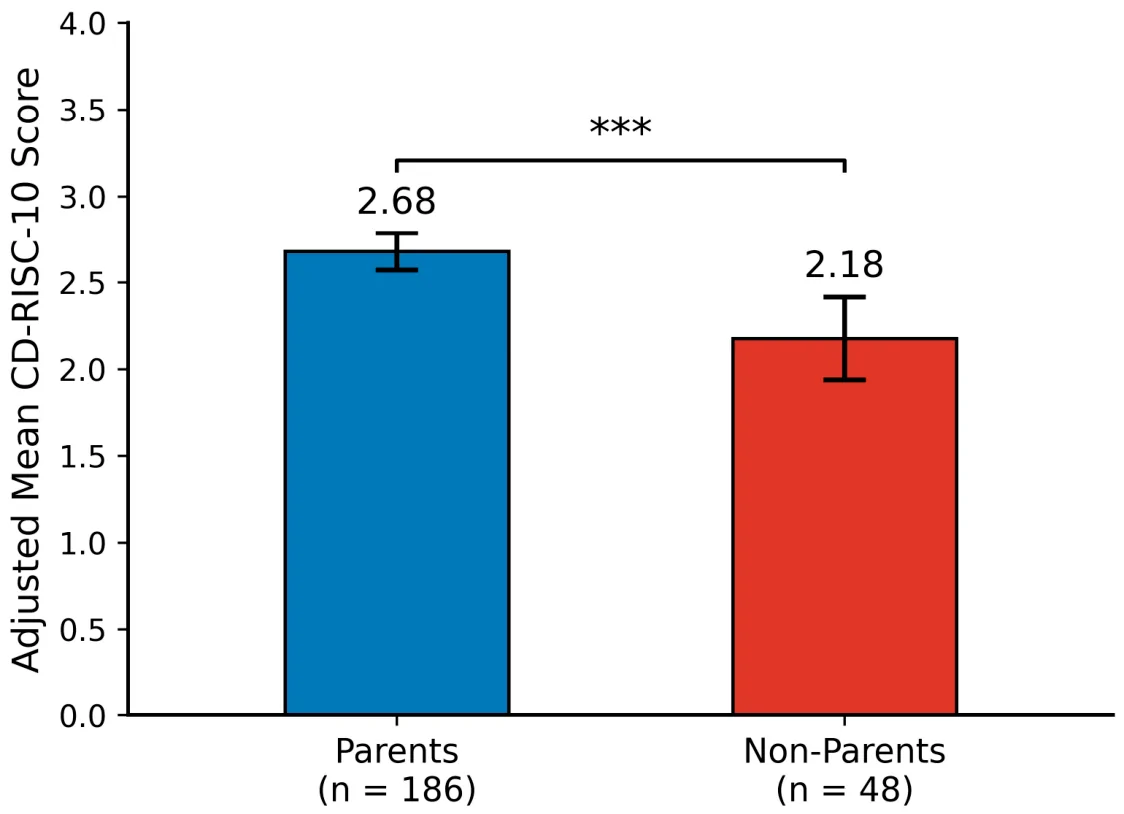
Adjusted CD-RISC-10 means by parenthood status (ANCOVA, N = 234). *Note:* ANCOVA adjusted for age, teaching experience, the five TIPI personality indicators, and marital status. Adjusted estimated marginal means: parents M_adj_ = 2.68, 95% CI [2.57, 2.79]; non-parents M_adj_ = 2.18, 95% CI [1.94, 2.42]; adjusted difference = 0.50, 95% CI [0.22, 0.78]; F(1, 224) = 12.65, *p* < 0.001. Error bars represent 95% confidence intervals. CD-RISC-10 scored as mean item score (range 0–4). This contrast is exploratory; see Section 3.4 and Table S1. Significance markers: *** *p* < 0.001.

A theory-guided ANCOVA, additionally adjusting for marital status beyond the primary regression covariates, yielded a directionally similar and statistically significant parenthood contrast, F(1, 224) = 12.65, *p* < 0.001, η^2^_p_ = 0.053. The full model is reported in Table S2. Adjusted estimated marginal means were 2.68 for parents (95% CI [2.57, 2.79]) and 2.18 for non-parents (95% CI [1.94, 2.42]), yielding an adjusted difference of 0.50, 95% CI [0.22, 0.78]. The adjusted marginal means are presented graphically in Figure 1. This contrast is exploratory and is displayed for transparency under severe covariate imbalance (only 27 of 186 parent cases were matchable 1:1 to the available non-parent controls) and should be read alongside the overlap diagnostics in Table S1 rather than as an adjusted estimate of a parenthood effect. The non-significance of marital status as an independent predictor in this model may reflect its association with parenthood (V = 0.33) and shared variance with age and teaching experience, rather than indicating the absence of substantive relevance; marital status is therefore treated as part of the broader life-stage configuration rather than as an independent explanatory factor.

### 3.5. Robustness Checks

Because the reported intervals assume constant error variance, the final model was re-estimated with HC3 heteroscedasticity-consistent standard errors (Table S9). The substantive pattern was unchanged. Emotional Stability (*p* < 0.001) and Conscientiousness (*p* < 0.001) remained the strongest observed dispositional correlates, and the parenthood coefficient remained statistically detectable, its standard error moving from 0.134 to 0.127 and its *p*-value remaining below 0.001. No coefficient changed significance status, and the largest change to any *p*-value in the model was 0.023. This is consistent with the residual diagnostics reported in Section 2.6.

Participant sex was not part of the primary specification for the reasons set out in Section 2.6, but because it is a plausible correlate of self-reported resilience in its own right, the final model was re-estimated with sex included (Table S10). The sex coefficient was small and non-significant (β = 0.02, *p* = 0.698); the model R^2^ moved from 0.410 to 0.410, and no other coefficient changed by more than 0.01 in standardized units. Including sex did not materially alter the pattern of results.

## 4. Discussion

This study documents observed variation in perceived resilience among educators who continued working under active wartime threat in Northern Israel between April and July 2024. Within the shared regional context defined by intensive missile threat, Emotional Stability and Conscientiousness were the strongest observed dispositional correlates of CD-RISC-10 scores, and parents reported higher scores than non-parents, a contrast that persisted across several analytic specifications without becoming separable from the life-stage characteristics with which parenthood co-varies. The Discussion distinguishes three kinds of statement throughout. Empirical findings are the observed associations reported in Section 3. Theoretical interpretations (Section 4.1 and Section 4.2) are frames within which those associations may be read; the psychological processes they invoke (emotion regulation, accumulated coping history, relational embeddedness, professional identity, and resource accumulation) were not measured in this study and are named as candidate explanations, not as documented processes. Practical implications (Section 4.4) follow from that interpretive reading rather than from the data directly, and are offered as testable directions rather than as recommendations established by these results.

Emotional Stability and Conscientiousness emerged as robust dispositional correlates of perceived resilience, in line with direct meta-analytic evidence on the link between Big Five personality traits and self-reported resilience (Oshio et al., 2018) and with systematic reviews of teacher resilience outcomes (Beltman et al., 2011; Mu et al., 2024; Munezero et al., 2026). These associations hold across multiple cultural contexts and occupational groups, providing relatively strong grounds for the claim that these dispositional traits are associated with perceived resilience under chronic threat. The meta-analytic evidence linking Big Five traits to perceived stress (Luo et al., 2023) further supports the broader theoretical picture, though perceived stress and perceived resilience are distinct outcomes.

At the same time, the association between Emotional Stability and perceived resilience should be read with measurement caution rather than as a purely dispositional effect. Emotional Stability (the low pole of Neuroticism) and self-reported resilience both index, in part, a tendency toward low negative affect and confidence in handling stress; so, their overlap may partly reflect shared conceptual content and a common self-report appraisal style. Because the TIPI Emotional Stability items and the CD-RISC-10 were completed within the same self-report session, common-method variance and a degree of criterion contamination cannot be excluded, and the observed coefficient (β = 0.37) may modestly overstate the independent contribution of disposition to perceived resilience; consistent with this concern, the two measures correlated r = 0.48 at the zero-order level, and re-estimating the model without Emotional Stability (Table S6) left the remaining associations, including Conscientiousness and the parenthood marker, essentially unchanged, indicating that the substantive conclusions do not hinge on this potentially overlapping predictor. We therefore interpret Emotional Stability as a correlate that is conceptually and methodologically entangled with the resilience outcome, not as an independently established causal determinant.

A complementary caution applies to the traits that did not predict perceived resilience. The present design yields positive, interpretable evidence for Emotional Stability and Conscientiousness, but the null results for Agreeableness, Openness, and Extraversion should not be read as evidence that these traits are unrelated to perceived resilience. Each was assessed with only two items, and the corresponding split-half reliabilities in this sample were low (Agreeableness SB = 0.58; Openness SB = 0.63; Extraversion SB = 0.65). Because low reliability attenuates observed associations and reduces statistical power, these null findings are uninformative rather than substantive: they are equally consistent with a genuine absence of association and with a true association obscured by measurement error. We therefore refrain from interpreting the non-significant personality coefficients and confine dispositional conclusions to the two traits measured with adequate, though still modest, reliability.

Recent teacher studies report that resilience co-varies with broader indicators of occupational functioning (Çiçek et al., 2026; Pöysä et al., 2026; Ren et al., 2025; Yang et al., 2025). Accordingly, the present results are best read as evidence of a resilience-related pattern within occupational functioning, not as evidence that one demographic characteristic is itself protective.

### 4.1. An Integrated Interpretation of the Psychological Correlates

The present findings may be interpreted not as three independent predictors of perceived resilience, but as a set of interrelated psychological correlates spanning dispositional, developmental, and contextual levels. At the dispositional level, Emotional Stability and Conscientiousness are dispositional tendencies that the broader literature has linked to adaptive functioning under sustained stress; the psychological processes underlying these associations were not measured here and are not inferred. At the developmental level, age, teaching experience, and parenthood co-vary and are read here as observable indicators of a broader life-stage position. The resources usually invoked to explain such a pattern (accumulated coping history, professional identity, relational embeddedness) were not measured in this study; they are named as candidate explanations for future testing, not as processes documented here. At the contextual level, all participants worked within the same wartime threat environment. We borrow the term ‘shared traumatic reality’ (Nuttman-Shwartz, 2015; Tosone et al., 2012) for one specific feature of that situation: the person carrying professional responsibility for others’ emotional containment is simultaneously exposed to the same danger; so, the professional and the personal cannot be held apart. The construct was developed for mental-health and welfare professionals treating trauma-exposed clients, and the extension to educators is deliberate and partial. Teachers are not trauma clinicians: they neither diagnose nor treat, and no equivalence of role, training, or clinical exposure is claimed. The extension claims only that the dual-role structure the construct names is also present in wartime teaching, and the term is used here as a descriptive label for that structure rather than as a tested proposition.

Interpreting these findings as an integrated correlate set, rather than as isolable determinants, is consistent with Conservation of Resources theory (Hobfoll, 1989, 2001; Hobfoll et al., 2018), which frames resilience as a function of the aggregated psychological resources available to an individual under stress rather than as the product of any single trait or demographic variable.

### 4.2. Life-Stage Configuration as an Interpretive Frame

Life-stage configuration is an interpretive frame, not a construct measured in this study. What was measured was a set of observed demographic characteristics: age, teaching experience, parenthood, and marital status. The developmental resources through which those characteristics are interpreted below (accumulated coping history, relational embeddedness, professional identity) were not assessed, and the step from one to the other is inferential rather than empirical. With that distinction in place, the frame aligns with the stress-appraisal model (Folkman, 2008; Lazarus & Folkman, 1984): individuals with longer coping histories and clearer occupational identity may appraise a broadly shared wartime context as more manageable, not because of parenthood but because of the full configuration of correlated characteristics in which parenthood is embedded. Reviews of teacher resilience and well-being report that career stage and professional experience are among the most consistent correlates of resilience-related outcomes in educational personnel (Beltman et al., 2011; Kurrle & Warwas, 2025; Lu et al., 2024; Mu et al., 2024). On this reading, demographic categories are poor proxies for psychological need, and direct assessment of resources and appraisal would be more informative (Southwick et al., 2014).

All participants worked within the same broad wartime context, but no individual-level exposure measure was collected; accordingly, the results describe associations within a shared regional context with unmeasured within-context variation (see Section 4.5).

The data do not support a causal claim that parenthood is psychologically protective, and neither do they establish that the difference is explained by age and teaching experience. What the overlap diagnostics show (Section 2.6 and Section 3.4; Table S1) is narrower: parenthood and life-stage position are statistically inseparable in this sample. The residual association surviving partial adjustment is therefore best described as attaching to an incompletely measured life-stage configuration. Its observed components include age, teaching experience, and marital status; unmeasured factors such as caregiving demands, resource history, professional seniority, relational embeddedness, and exposure heterogeneity may also contribute. The present design cannot adjudicate among these components, and the observed difference should not be reported as having been accounted for by any subset of them.

### 4.3. Methodological Strengths and Broader Relevance

Several features strengthen this study despite its inferential limits. First, the sample was gathered during an actual period of sustained missile threat rather than through retrospective reconstruction. Second, this study focuses on educators, a group whose psychological functioning matters not only for their own well-being but also for continuity of teaching and student support under crisis conditions. Third, the analysis does not attempt to rescue a weak comparison with overconfident statistical language; instead, it makes the subgroup comparison transparent by reporting overlap diagnostics, sensitivity analyses, and explicit limits on causal interpretation.

These features also influence what this study contributes beyond the immediate setting. The findings add dispositional-resilience evidence from an Israeli educator population under active missile threat, a group underrepresented in the resilience literature, and thereby allow personality–resilience associations established largely in Western, non-conflict samples to be examined in a cultural and threat context rarely represented in this literature. The transparency of the analytic approach (overlap diagnostics and stated limits in place of an adjusted contrast presented as though balance had been achieved) may also be useful for other field research on structurally non-overlapping groups.

The observed effect size also deserves careful interpretation. A raw subgroup contrast of d = 0.68 is not negligible, and it would be easy, but misleading, to convert that finding into a simple narrative about parenthood as protection. The more defensible reading is the narrower one set out above: real variation existed within this workforce, and parenthood marks where it fell.

### 4.4. Cautious Implications and Future Research Priorities

As set out in Section 4.2, the observed variation is read here as marking a life-stage position rather than a parenthood effect (Grundström et al., 2024; Hobfoll et al., 2018; Nomaguchi & Milkie, 2003).

Because no stress, coping, or intervention processes were measured here, any applied implications are necessarily tentative and contingent on replication in stronger designs. With that caveat, the prominence of Emotional Stability and Conscientiousness, rather than demographic status, suggests a modest and testable direction for future work: directly assessing and supporting candidate psychological resources (not assessed in the present study) across the whole educator workforce may be more informative than treating demographic categories such as parenthood as proxies for psychological need (Beltman et al., 2011; Mansfield et al., 2016). This direction is consistent with work on emotion regulation as a moderator of war-exposure effects on teacher burnout (Neustadter et al., 2026), with multi-level models of educator support (Patrick et al., 2024; Tarrasch & Russo-Netzer, 2026), and with evidence that structured support can be delivered to comparable professions during continued adversity (Berger & Gelkopf, 2011; Harb et al., 2026; Täschner et al., 2025). Whether such approaches actually improve perceived resilience among educators, however, remains an empirical question for future longitudinal and intervention research rather than a conclusion of the present cross-sectional data.

### 4.5. Limitations and Future Directions

Several limitations constrain the strength of inference that can be drawn from the present study. The cross-sectional design precludes causal or temporal conclusions. Parenthood was a blunt binary indicator, with no data on number or ages of children, caregiving intensity, single-parent status, or the psychological demands of parenting during wartime. The covariate imbalance documented in Section 3.2 cannot be resolved analytically with these data and remains the most important interpretive constraint (Ho et al., 2007; Stuart, 2010).

No direct measures of perceived stress, coping strategies, social support, trauma-related symptoms, or individual-level war-exposure severity were included. This study therefore addresses perceived resilience as a cross-sectional outcome rather than the processes producing it. All participants shared an inclusion-defining context of active missile threat in Northern Israel, but the data do not distinguish between the broadly shared regional context and finer gradations of exposure intensity within the sample. Because individual exposure was not measured, the extent to which finer differences in threat exposure contributed to the observed variation in perceived resilience cannot be determined, and unmeasured variation in exposure may have confounded any of the reported associations, not only the parenthood contrast. Future studies should incorporate ethically appropriate indicators of individual exposure (evacuation or displacement history, duration of school closure, perceived threat, and regional attack intensity) so that within-context variation can be modeled rather than assumed. Convenience sampling through institutional gatekeepers without a calculable response rate further limits representativeness and should caution against extrapolation beyond similar field settings. In addition, although post hoc response-quality screening (long string, within-person response variability, and reverse-keyed consistency) (Curran, 2016; Meade & Craig, 2012) flagged 16 cases (6.8%) and the results were robust to their exclusion (n = 218; Table S7), the anonymous administration precluded verification against IP addresses or completion-time metadata; so, a residual possibility of undetected duplicate or careless responding cannot be entirely excluded.

The unusually high internal consistency of the CD-RISC-10 (α = 0.97) may reflect a highly homogeneous item set and/or shared method variance (Podsakoff et al., 2003). Relatedly, because Emotional Stability and the resilience outcome were both self-reported within a single session and overlap conceptually, the Emotional Stability–resilience association may be partly inflated by common-method variance and criterion contamination, as detailed at the start of Section 4 (zero-order r = 0.48; see Table S6 for the model re-estimated without Emotional Stability). Measurement invariance across subgroups was not formally tested (Putnick & Bornstein, 2016). The marginal reliability of Agreeableness (SB = 0.58) may also attenuate its regression coefficient (Credé et al., 2012).

A further limitation concerns the structure of the data rather than their content. Educators are nested within schools, and schools differ in organizational climate, leadership, institutional resources, emergency procedures, and local exposure to attack. Because the survey was anonymous and school identifiers were not collected, this hierarchical structure could not be modeled, and two consequences follow. First, if resilience scores are correlated within schools, the standard errors reported here are likely to be underestimated and the associated *p*-values correspondingly optimistic; the significance levels in Table 4 and Table 5 should be read with that in mind. Second, individual-level and organizational-level sources of variation cannot be separated; so, an association attributed here to a teacher characteristic may partly reflect the school in which that teacher works. Future studies in this setting should collect school identifiers alongside school-level variables (leadership, perceived organizational support, closure duration, local attack intensity) and estimate multilevel models capable of decomposing variance into individual and organizational components (Raudenbush & Bryk, 2002).

Three analyses added at revision bear on the robustness of what is reported here rather than on its scope. HC3 re-estimation left every coefficient’s significance unchanged (Section 3.5), a one-factor confirmatory factor analysis supported the unidimensional scoring of the CD-RISC-10 (Section 2.5), and a sex-inclusive specification left the pattern intact (Section 3.5). None of the three addresses this study’s principal limitations, which remain the absence of an individual exposure measure, the cross-sectional design, and the unmodeled school structure.

Future research should adopt longitudinal or repeated-measure designs with direct measurement of war-exposure severity, perceived stress, coping strategies, social support, resource inventories, and trauma-related symptoms. Future studies should also examine community-level moderators of resilience directly (including social support, intergenerational ties, and school-community resources) rather than inferring such processes from the sample. Multilevel designs incorporating school-level clustering would allow for more precise decomposition of individual, relational, and organizational sources of variance. Randomized or quasi-experimental studies of educator emotion-regulation support under conflict conditions are needed to test whether the correlates identified here can be translated into trainable protective processes.

Future studies would also benefit from integrating educator well-being implementation frameworks with conflict-zone intervention designs so that resilience assessment, organizational support planning, and brief scalable supports can be evaluated together rather than in isolation. Such work would help move the field from transparent descriptive reporting toward mechanism-sensitive prevention and intervention research.

## 5. Conclusions

In a sample of 234 educational staff who continued working under active wartime missile threat between April and July 2024, perceived resilience was associated primarily with Emotional Stability and Conscientiousness. The exploratory parent–non-parent contrast co-occurred with an incompletely measured demographic and life-stage configuration and should not be interpreted as an independent effect of parenthood. Age and teaching experience are too highly correlated in this sample (r = 0.82) to be separated: each reaches significance when entered alone, and neither survives when both are entered (*p* = 0.593 and *p* = 0.155); so, they are reported as joint indicators of a single dimension and not as independently established correlates. Emotional Stability, likewise, is reported as the strongest observed dispositional correlate rather than as an independent predictor, given its conceptual and method overlap with the resilience measure. Consistent with this study’s descriptive aims, the overall pattern is presented as observed variation in perceived resilience rather than as evidence for any single explanatory variable, and parenthood is treated throughout as an exploratory marker. The higher scores among parent educators are not shown to be explained by age and experience; they attach to an incompletely measured life-stage configuration that these data cannot decompose.

Taken together, the findings describe patterned variation in perceived resilience across dispositional, demographic, and contextual correlates within a shared regional threat context whose individual-level intensity was not measured. Practically, and as an implication of the interpretive frame rather than a direct result, the findings favor assessing current strain and support needs over demographic status when planning educator support. The observed associations identify personality-related correlates of perceived resilience; they do not establish emotion regulation or self-regulation as measured mechanisms, nor test whether these processes are modifiable. By examining whether well-established personality–resilience associations are also observed among educators working under sustained regional missile threat, this study offers a contextual extension of that literature to a population rarely represented in it, and a foundation for the longitudinal and intervention research that questions of mechanism and modifiability require.

## Figures and Tables

**Table 1:** Participant characteristics by parenthood status (parents vs. non-parents; N = 234).

Variable	Full Sample (N = 234)	Parents (n = 186)	Non-Parents (n = 48)	Effect Size	*p*
Age, M (SD)	42.5 (9.0)	44.8 (7.8)	33.7 (7.7)	d = 1.41	<0.001
Teaching Experience, M (SD)	17.4 (8.0)	19.2 (7.5)	10.6 (6.1)	d = 1.18	<0.001
Male, n (%)	101 (43.2%)	79 (42.5%)	22 (45.8%)	—	—
Female, n (%)	133 (56.8%)	107 (57.5%)	26 (54.2%)	χ^2^(1) = 0.18	0.675
Married/Cohabiting, n (%)	191 (81.6%)	164 (88.2%)	27 (56.3%)	V = 0.33	<0.001
CD-RISC-10, M (SD)	2.58 (0.90)	2.70 (0.85)	2.11 (0.92)	d = 0.68 ***	<0.001

*Note:* M = mean; SD = standard deviation. Effect sizes: Cohen’s d for continuous variables; Cramér’s V for categorical. CD-RISC-10 scored as mean item score (range 0–4). χ^2^(1) = 0.18 for sex (male vs. female) (*p* = 0.675). Significance markers: *** *p* < 0.001.

**Table 2:** Descriptive statistics and internal consistency for outcome and personality measures (N = 234).

Variable	M	SD	Min	Max	Reliability
CD-RISC-10 (Perceived Resilience)	2.58	0.90	0.00	4.00	α = 0.97
Extraversion	4.18	1.40	1.00	7.00	SB = 0.65
Agreeableness	5.09	1.16	1.50	7.00	SB = 0.58
Conscientiousness	5.31	1.12	1.50	7.00	SB = 0.71
Emotional Stability	4.85	1.25	1.50	7.00	SB = 0.74
Openness	4.66	1.23	1.00	7.00	SB = 0.63

*Note:* CD-RISC-10 = Connor–Davidson Resilience Scale (10-item version; range 0–4); α = Cronbach’s alpha. TIPI variables (Gosling et al., 2003) measured on 7-point scale; SB = Spearman–Brown split-half reliability for two-item subscales (Credé et al., 2012).

**Table 3:** Zero-order correlations among continuous study variables (N = 234).

Variable	1	2	3	4	5	6	7	8
1. CD-RISC-10	—							
2. Age	0.36 ***	—						
3. Teaching experience	0.35 ***	0.82 ***	—					
4. Extraversion	0.18 **	−0.05	−0.04	—				
5. Agreeableness	0.16 *	0.10	0.09	0.20 **	—			
6. Conscientiousness	0.38 ***	0.18 **	0.17 **	0.15 *	0.28 ***	—		
7. Emotional stability	0.48 ***	0.16 *	0.14 *	0.22 ***	0.24 ***	0.30 ***	—	
8. Openness	0.17 **	−0.02	−0.03	0.25 ***	0.18 **	0.16 *	0.19 **	—

*Note:* Pearson’s correlations. CD-RISC-10 = Connor–Davidson Resilience Scale (10-item version); Big Five indicators from the TIPI (Gosling et al., 2003). Parenthood status, a dichotomous exploratory marker, is not included here and is analyzed via the group comparison (Table 1) and the hierarchical model (Table 4). Approximate critical values for N = 234 (two-tailed): |r| ≥ 0.13, *p* < 0.05; |r| ≥ 0.17, *p* < 0.01; |r| ≥ 0.21, *p* < 0.001. * *p* < 0.05; ** *p* < 0.01; *** *p* < 0.001.

**Table 4:** Hierarchical multiple regression predicting CD-RISC-10 scores (N = 234).

Predictor	B	SE	β	t	*p*	95% CI
*Model 1 (Step 1): Age and Teaching Experience*						
Constant	1.31	0.31	—	4.19	<0.001	[0.69, 1.92]
Age	0.022	0.011	0.22 *	2.09	0.038	[0.001, 0.043]
Teaching Experience	0.019	0.012	0.17	1.57	0.118	[−0.005, 0.042]
*Model 1 fit: R*^2^ *= 0.139, Adj. R*^2^ *= 0.131, F(2, 231) = 18.61, p < 0.001*						
*Model 2 (Step 2): + Big Five Personality Indicators*						
Constant	−0.76	0.39	—	−1.92	0.056	[−1.53, 0.02]
Age	0.015	0.009	0.15	1.67	0.097	[−0.003, 0.034]
Teaching Experience	0.016	0.010	0.15	1.60	0.112	[−0.004, 0.037]
Extraversion	0.05	0.04	0.08	1.36	0.175	[−0.02, 0.12]
Agreeableness	−0.03	0.04	−0.04	−0.69	0.490	[−0.12, 0.06]
Conscientiousness	0.17	0.05	0.21 ***	3.67	<0.001	[0.08, 0.26]
Emotional Stability	0.25	0.04	0.35 ***	6.12	<0.001	[0.17, 0.33]
Openness	0.05	0.04	0.06	1.17	0.243	[−0.03, 0.13]
*Model 2 fit: R*^2^ *= 0.374, Adj. R*^2^ *= 0.355, ΔR*^2^ *= 0.236, ΔF(5, 226) = 17.03, F(7, 226) = 19.32, p < 0.001*						
*Model 3 (Step 3): + Parenthood Status (Life-Stage Marker)*						
Constant	−0.89	0.38	—	−2.31	0.022	[−1.64, −0.13]
Age	0.005	0.009	0.05	0.54	0.593	[−0.014, 0.024]
Teaching Experience	0.014	0.010	0.13	1.43	0.155	[−0.005, 0.034]
Extraversion	0.05	0.03	0.07	1.35	0.177	[−0.02, 0.12]
Agreeableness	−0.02	0.04	−0.02	−0.42	0.671	[−0.10, 0.07]
Conscientiousness	0.19	0.05	0.24 ***	4.19	<0.001	[0.10, 0.28]
Emotional Stability	0.27	0.04	0.37 ***	6.59	<0.001	[0.19, 0.35]
Openness	0.05	0.04	0.06	1.16	0.249	[−0.03, 0.12]
Parenthood Status	0.49	0.13	0.22 ***	3.66	<0.001	[0.23, 0.76]
*Model 3 fit: R*^2^ *= 0.410, Adj. R*^2^ *= 0.389, ΔR*^2^ *= 0.035, ΔF(1, 225) = 13.41, F(8, 225) = 19.51, p < 0.001*						

*Note:* CD-RISC-10 = Connor–Davidson Resilience Scale (10-item version). B = unstandardized coefficient; SE = standard error; β = standardized coefficient. Parenthood Status coded 1 = parent, 0 = non-parent. Coefficients are reported separately for each model; model-fit statistics are cumulative. Constant is the unstandardized intercept; because predictors are uncentered, it lies outside the 0–4 CD-RISC-10 range. Confidence intervals are 95% OLS intervals; Age and Teaching Experience are displayed to three decimal places throughout and the remaining predictors to two. *p*-values reported to three decimals when *p* ≥ 0.001 and as *p* < 0.001 otherwise. Asterisks appear on β only. VIF ≤ 3.4 for all predictors. Step 3 is exploratory (Section 2.6). Significance markers: * *p* < 0.05; *** *p* < 0.001.

**Table 5:** Stability of the parenthood coefficient across life-stage covariate specifications (N = 234).

Life-Stage Specification	Parenthood B (SE)	Parenthood β	Parenthood *p*	Parenthood 95% CI	Age β	Experience β	Model R^2^
Age only	0.50 (0.13)	0.23	<0.001	[0.24, 0.77]	0.15 *	—	0.404
Teaching experience only	0.51 (0.13)	0.23	<0.001	[0.26, 0.76]	—	0.16 **	0.409
Age + experience (final model)	0.49 (0.13)	0.22	<0.001	[0.23, 0.76]	0.05	0.13	0.410

*Note:* All three models additionally include the five TIPI personality indicators (coefficients omitted for brevity). * *p* < 0.05; ** *p* < 0.01.

## Data Availability

The data presented in this study are available on request from the corresponding author due to ethical and privacy restrictions related to the small wartime field sample and the risk of participant re-identification. Requests will be considered subject to the applicable ethics and confidentiality safeguards. All aggregate results needed to evaluate the conclusions of this study (including descriptive statistics, hierarchical regression results, and sensitivity analyses) are reported within this article and its Supplementary Materials.

## Supplementary Materials

The following supporting information can be downloaded at: https://www.mdpi.com/article/10.3390/bs16091615/s1, Table S1, Propensity Score Matching (PSM) Diagnostics: Matching Outcome and Covariate Balance (N = 234); Table S2, Sensitivity ANCOVA Additionally Adjusting for Marital Status (N = 234); Table S3, Non-Parametric Robustness Test for the CD-RISC-10 Group Comparison (N = 234); Table S4, Age-Restricted Subgroup Descriptives (ages 23–47 years; n = 160); Table S5, Independent-Sample t-Test for the Age-Restricted Subgroup (n = 160); Table S6, Hierarchical Regression Predicting CD-RISC-10 Scores with Emotional Stability Removed (N = 234); Table S7, Response-Quality (Careless-Responding) Screening and Sensitivity Analysis (N = 234); Table S8, Confirmatory Factor Analysis of the CD-RISC-10: One-Factor Model (N = 234); Table S9, Final Hierarchical Model with HC3 Heteroscedasticity-Consistent Standard Errors (N = 234); Table S10, Final Model Re-Estimated with Participant Sex Included (N = 234); and Figure S1, Unadjusted Group Means for CD-RISC-10 by Parenthood Status. All supplementary tables and the supplementary figure were computed from the same analysis file as the results reported in the main text and correspond to the present version of this manuscript. Written permission from the copyright holder for use of the Hebrew CD-RISC-10 was obtained and is available from the corresponding author upon request.

## Abbreviations

The following abbreviations are used in this manuscript: ANCOVA, analysis of covariance; CD-RISC-10, Connor–Davidson Resilience Scale (10-item version); CI, confidence interval; COR, Conservation of Resources theory; IRB, institutional review board; JARS, journal article reporting standards; OLS, ordinary least squares; PSM, propensity score matching; SB, Spearman–Brown reliability coefficient; SMD, standardized mean difference; STROBE, strengthening the reporting of observational studies in epidemiology; TIPI, Ten-Item Personality Inventory; VIF, variance inflation factor.
