# Supplementary material for: Teaching Under Fire: A Cross-Sectional Study of Dispositional and Life-Stage Correlates of Perceived Resilience Among Israeli Educators Under Missile Threat
Source: Behav Sci (Basel). 2026 Sep 10;16(9):1615. doi: 10.3390/bs16091615 (PMC13603468; doi:10.3390/bs16091615)
Supplement: Supplementary file 1 [file behavsci-16-01615-s001.zip › behavsci-4449118-supplementary.pdf]

Supplementary Materials

Supplementary materials for: Teaching Under Fire: A Cross-Sectional Study of Dispositional and Life-Stage Correlates of Perceived Resilience Among Israeli Educators Under Missile Threat

Contains: Tables S1–S10 and Figure S1. All tables were computed from the same analysis file as the main manuscript. Tables S8–S10 were added at revision in response to the reviewers.

Table S1

Propensity Score Matching (PSM) Diagnostics: Matching Outcome and Covariate Balance (N = 234)

PSM diagnostic	Before matching	After matching	Interpretation
Total parent cases	n = 186	n = 27	159 (85.5%) unmatched
Total non-parent cases	n = 48	n = 27	21 unmatched, outside caliper
Caliper	—	0.20 SD logit	Nearest-neighbor, without replacement
SMD: Age	1.42	−0.01	Reduced after matching
SMD: Teaching Experience	1.25	−0.18	Reduced after matching
SMD: Married	0.76	−0.16	Reduced after matching
SMD: Extraversion	−0.16	0.40	Not improved
SMD: Agreeableness	−0.16	−0.13	Reduced after matching
SMD: Conscientiousness	−0.14	−0.28	Not improved
SMD: Emotional Stability	−0.11	0.15	Not improved
SMD: Openness	−0.11	0.26	Not improved

Note: Propensity scores were estimated by logistic regression of parenthood status on age, teaching experience, marital status and the five personality indicators, then matched 1:1 by nearest neighbor within a caliper of 0.20 SD of the logit. SMD = standardized mean difference, computed with the square root of the average of the two group variances in the denominator, as is conventional in matching diagnostics; the effect sizes in Table 1 use the pooled standard deviation, so the two differ slightly for the same variable. Only 27 of 186 parent cases could be matched, so the matched analysis is reported for transparency about overlap rather than as an adjusted estimate.

Table S2

Sensitivity ANCOVA Additionally Adjusting for Marital Status (N = 234)

Predictor	B	SE	β	t	p	95% CI
Age	0.005	0.009	0.05	0.51	0.611	[−0.014, 0.024]
Teaching Experience	0.014	0.010	0.13	1.44	0.152	[−0.005, 0.034]
Extraversion	0.05	0.03	0.07	1.36	0.175	[−0.02, 0.12]
Agreeableness	−0.02	0.04	−0.02	−0.41	0.684	[−0.10, 0.07]
Conscientiousness	0.19	0.05	0.24***	4.17	<0.001	[0.10, 0.28]
Emotional Stability	0.27	0.04	0.37***	6.56	<0.001	[0.19, 0.34]
Openness	0.05	0.04	0.06	1.16	0.248	[−0.03, 0.12]
Marital Status	−0.03	0.13	−0.01	−0.25	0.802	[−0.28, 0.22]
Parenthood Status	0.50	0.14	0.23***	3.56	<0.001	[0.22, 0.78]

*Note: Model fit: $R^2 = 0.410$, adjusted $R^2 = 0.386$, $F(9, 224) = 17.28$, $p < 0.001$. The parenthood contrast is $F(1, 224) = 12.65$, $p < 0.001$, $\eta^2_p = 0.053$; adjusted marginal means 2.68 for parents and 2.18 for non-parents. Marital status coded 1 = married/cohabiting; Parenthood Status coded 1 = parent. *** $p < 0.001$.*

Table S3

Non-Parametric Robustness Test for the CD-RISC-10 Group Comparison (N = 234)

Test	Statistic	z	p	Effect size
Mann–Whitney U	6057	3.81	<0.001	r = 0.25
Welch's t	t(69.6) = 4.04	—	<0.001	d = 0.68

Note: The parametric and non-parametric tests agree in direction and significance, so the unadjusted group difference does not depend on the normality assumption. Effect size r for the Mann–Whitney test is |z| / √N.

Table S4

Age-Restricted Subgroup Descriptives (ages 23–47 years; n = 160)

Variable	Parents (n = 118)	Non-Parents (n = 42)
Age, M (SD)	40.4 (5.1)	33.3 (6.0)
Teaching Experience, M (SD)	16.1 (6.5)	10.6 (5.5)
CD-RISC-10, M (SD)	2.59 (0.85)	2.07 (0.93)

Note: The subgroup restricts both groups to the age range spanned by the middle 90% of non-parents (23–47 years), which is the region of the age distribution where the two groups overlap.

Table S5

Independent-Sample t-Test for the Age-Restricted Subgroup (n = 160)

Comparison	t	df	p	Cohen's d
CD-RISC-10, parents vs. non-parents	3.25	67.4	0.002	0.61

Note: Welch's correction applied. Restricting the age range reduces the covariate imbalance but also the available sample, so this comparison is reported as a robustness check rather than as the primary estimate.

Table S6

Hierarchical Regression Predicting CD-RISC-10 Scores with Emotional Stability Removed (N = 234)

Predictor	B	SE	β	t	p	95% CI
Age	0.011	0.010	0.11	1.05	0.296	[−0.009, 0.031]
Teaching Experience	0.015	0.011	0.14	1.39	0.166	[−0.006, 0.037]
Extraversion	0.08	0.04	0.13*	2.17	0.031	[0.01, 0.16]
Agreeableness	0.01	0.05	0.02	0.32	0.748	[−0.08, 0.11]
Conscientiousness	0.25	0.05	0.31***	5.10	<0.001	[0.15, 0.34]
Openness	0.07	0.04	0.10	1.71	0.089	[−0.01, 0.16]
Parenthood Status	0.41	0.15	0.19**	2.83	0.005	[0.12, 0.70]

*Note: This model re-estimates the final hierarchical regression with the Emotional Stability indicator removed, addressing the concern that Emotional Stability (the low pole of Neuroticism) and the CD-RISC-10 may share construct and method variance (their zero-order correlation was r = 0.48; see Table 3 and the opening of Section 4 of the main text). Removing Emotional Stability reduced the explained variance from $R^2 = 0.410$ (full model, Table 4) to $R^2 = 0.296$, a decrement of $\Delta R^2 = 0.114$ attributable uniquely to Emotional Stability. The remaining associations were essentially unchanged: Conscientiousness (β = 0.31) and the parenthood marker (β = 0.19) retained their magnitude and significance. The substantive conclusions therefore do not depend on the potentially overlapping Emotional Stability predictor. Model fit: $R^2 = 0.296$, adjusted $R^2 = 0.274$, $F(7, 226) = 13.55$, $p < 0.001$. Parenthood Status coded 1 = parent, 0 = non-parent. * $p < 0.05$; ** $p < 0.01$; *** $p < 0.001$.*

Table S7

Response-Quality (Careless-Responding) Screening and Sensitivity Analysis (N = 234)

Screening criterion	Operational threshold	n flagged (%)
Long string index across the 20 CD-RISC-10 and TIPI items	> 10 identical consecutive responses	1 (0.4%)
Intra-individual response variability on the CD-RISC-10	Within-person SD = 0	16 (6.8%)
TIPI within-domain consistency	Discrepancy > 3 points on ≥ 3 of the five domains	0 (0.0%)
Flagged by at least one criterion (union)	—	16 (6.8%)

Note: Post hoc careless-responding screening followed established procedures (Curran, 2016; Meade & Craig, 2012). Because forced-response administration produced complete data, no participant could be flagged for missingness; screening therefore targeted low-variability and inconsistent responding. A sensitivity analysis excluding the flagged cases (n = 218) reproduced the pattern of results: Emotional Stability ($\beta = 0.36$, $p < 0.001$), Conscientiousness ($\beta = 0.22$, $p < 0.001$) and the parenthood marker ($\beta = 0.20$, $p = 0.002$) were essentially unchanged, and total model fit was $R^2 = 0.370$. All 234 cases were therefore retained in the analyses reported in the main text.

Table S8

Confirmatory Factor Analysis of the CD-RISC-10: One-Factor Model (N = 234)

Index	Value	Conventional criterion	Assessment
χ^2 (df = 35)	46.01	Non-significant preferred	p = 0.101, ns
CFI	0.996	≥ 0.95	Good
TLI	0.995	≥ 0.95	Good
RMSEA	0.037	≤ 0.06	Good
SRMR	0.013	≤ 0.08	Good
Standardized loadings	0.84–0.91	≥ 0.40	All items load strongly
McDonald's ω	0.97	≥ 0.70	High; interpreted with the item-redundancy caveat (see note)
Cronbach's α	0.97	≥ 0.70	High; interpreted with the item-redundancy caveat (see note)
Average inter-item r	0.77	0.15–0.50	Above range — item redundancy

Note: Maximum-likelihood estimation on the ten CD-RISC-10 items. The one-factor solution fits well, supporting the unidimensional mean-score scoring used throughout the manuscript. The average inter-item correlation lies above the 0.15–0.50 band described by Clark and Watson (1995) as indicating adequate but non-redundant item coverage. McDonald's ω was essentially identical to α. Together with the average inter-item correlation, these coefficients indicate high internal consistency and substantial item overlap; the average inter-item correlation is the index that speaks directly to redundancy.

Table S9

Final Hierarchical Model with HC3 Heteroscedasticity-Consistent Standard Errors (N = 234)

Predictor	B	OLS SE	HC3 SE	t (HC3)	p (HC3)	95% CI (HC3)
Age	0.005	0.009	0.009	0.56	0.575	[−0.013, 0.023]
Teaching Experience	0.014	0.010	0.011	1.35	0.178	[−0.007, 0.035]
Extraversion	0.05	0.03	0.04	1.30	0.195	[−0.02, 0.12]
Agreeableness	−0.02	0.04	0.04	−0.41	0.685	[−0.11, 0.07]
Conscientiousness	0.19	0.05	0.05	3.92	<0.001	[0.09, 0.28]
Emotional Stability	0.27	0.04	0.04	6.40	<0.001	[0.18, 0.35]
Openness	0.05	0.04	0.04	1.18	0.240	[−0.03, 0.12]
Parenthood Status	0.49	0.13	0.13	3.88	<0.001	[0.24, 0.74]

Note: HC3 heteroscedasticity-consistent (robust) standard errors for the final model (Model 3) reported in Table 4 of the main text. Point estimates, and therefore the model fit ($R^2 = 0.410$, adjusted $R^2 = 0.389$), are unchanged by construction; only the standard errors, and with them the test statistics and intervals, differ. Age and Teaching Experience are displayed to three decimal places and the remaining predictors to two. No coefficient changed significance status, and the largest change to any p-value was 0.023. The parenthood coefficient remains statistically detectable ($p < 0.001$), as do Emotional Stability and Conscientiousness. Parenthood Status coded 1 = parent, 0 = non-parent.

Table S10

Final Model Re-Estimated with Participant Sex Included (N = 234)

Predictor	B	SE	β	t	p
Age	0.005	0.009	0.05	0.52	0.605
Teaching Experience	0.014	0.010	0.13	1.41	0.161
Extraversion	0.05	0.04	0.07	1.30	0.196
Agreeableness	−0.02	0.04	−0.02	−0.41	0.682
Conscientiousness	0.19	0.05	0.24***	4.19	<0.001
Emotional Stability	0.27	0.04	0.37***	6.56	<0.001
Openness	0.05	0.04	0.06	1.16	0.249
Parenthood Status	0.49	0.13	0.22***	3.66	<0.001
Sex (1 = female)	0.04	0.09	0.02	0.39	0.698

*Note: Sex coded 1 = female, 0 = male. Age and Teaching Experience are displayed to three decimal places and the remaining predictors to two. Extraversion's standard error falls almost exactly on the two-decimal rounding boundary: it rounds down to 0.03 in the final model (Table 4 of the main text, and the OLS column of Table S9) and up to 0.04 here, once sex is added. The difference between the two is immaterial; Extraversion's β (0.07) and its non-significance are the same in both models. Model fit: $R^2 = 0.410$, adjusted $R^2 = 0.386$, $F(9, 224) = 17.30$, $p < 0.001$, against $R^2 = 0.410$ and adjusted $R^2 = 0.389$ for the model without sex (Table 4 of the main text). The sex coefficient is small and non-significant (β = 0.02, $p = 0.698$), and the substantive pattern is unaffected. *** $p < 0.001$.*

Figure S1

Unadjusted Group Means for CD-RISC-10 by Parenthood Status

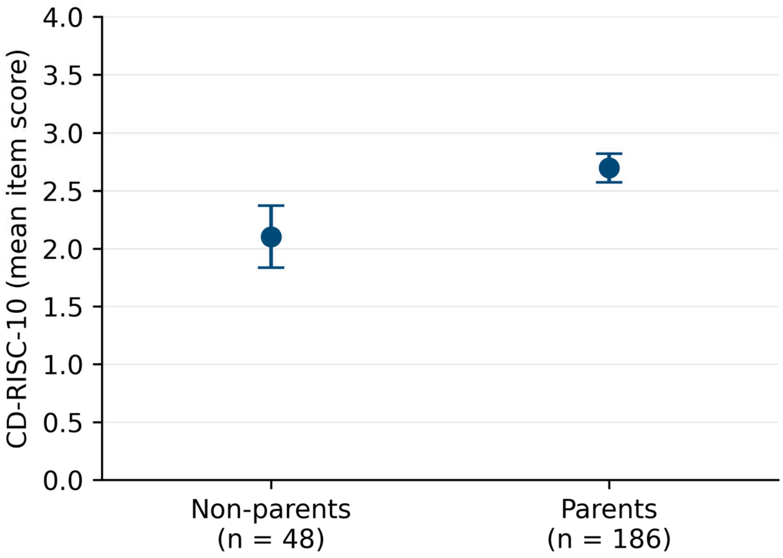

Note: Unadjusted means with 95% confidence intervals based on the t distribution; N = 234. Non-parents M = 2.11, 95% CI [1.84, 2.37]; parents M = 2.70, 95% CI [2.57, 2.82]. This figure shows the raw contrast before any covariate adjustment; the adjusted means are given in Figure 1 of the main text. The groups differ substantially in age and teaching experience (Table 1), so the unadjusted difference should not be read as an effect of parenthood.
